# Latitudinal and temporal variation in injury and its impacts in the invasive Asian shore crab *Hemigrapsus sanguineus*

**DOI:** 10.1038/s41598-022-21119-1

**Published:** 2022-10-03

**Authors:** Blaine D. Griffen, Jill Alder, Lars Anderson, Emily Gail Asay, April Blakeslee, Mikayla Bolander, Doreen Cabrera, Jade Carver, Laura C. Crane, Eleanor R. DiNuzzo, Laura S. Fletcher, Johanna Luckett, Morgan Meidell, Emily Pinkston, Tanner C. Reese, Michele F. Repetto, Nanette Smith, Carter Stancil, Carolyn K. Tepolt, Benjamin J. Toscano, Ashley Vernier

**Affiliations:** 1 Department of Biology, Brigham Young University, Provo, UT 84602 USA; 2 Biology Department, Southern Utah University, Cedar City, UT 84720 USA; 3 Department of Biology, East Carolina University, Greenville, NC 27858 USA; 4 Department of Biology, Temple University, Philadelphia, PA 19122 USA; 5 Department of Biology, Woods Hole Oceanographic Institution, Woods Hole, MA 02543 USA; 6 Department of Biology, Trinity College, Hartford, CT 06106 USA; 7 Wells National Estuarine Research Reserve, Wells, ME 04090 USA

**Keywords:** Ecophysiology, Invasive species

## Abstract

Nonlethal injury is a pervasive stress on individual animals that can affect large portions of a population at any given time. Yet most studies examine snapshots of injury at a single place and time, making the implicit assumption that the impacts of nonlethal injury are constant. We sampled Asian shore crabs *Hemigrapsus sanguineus* throughout their invasive North American range and from the spring through fall of 2020. We then documented the prevalence of limb loss over this space and time. We further examined the impacts of limb loss and limb regeneration on food consumption, growth, reproduction, and energy storage. We show that injury differed substantially across sites and was most common towards the southern part of their invaded range on the East Coast of North America. Injury also varied idiosyncratically across sites and through time. It also had strong impacts on individuals via reduced growth and reproduction, despite increased food consumption in injured crabs. Given the high prevalence of nonlethal injury in this species, these negative impacts of injury on individual animals likely scale up to influence population level processes (e.g., population growth), and may be one factor acting against the widespread success of this invader.

## Introduction

Nonlethal injury and regeneration of lost body parts are very common across both vertebrate and invertebrate animals^1–3^. While the frequency of injury varies across species and populations, on average more than 25% of individual animals within populations are dealing with injuries at any given time^4^. This number is likely an underestimate because some immediately nonlethal injuries can reduce future survival by increasing future predation risk, thereby disproportionately removing these individuals from populations^5^. Further, the frequency of injury in animal populations is likely to rise moving forward because animal injury rates generally increase with human presence^6–8^. Consequently, accurately assessing population growth and stability requires incorporating the (possibly variable) negative consequences of nonlethal injury for individual growth and reproduction.

Existing studies on nonlethal injury often document its occurrence at a single location and moment in time and infer implications for population dynamics from this limited information. Nonlethal injury can, however, vary for a single species across spatial and temporal scales^9^. Yet, there is little information on this variation for most species, with the result that the influence of nonlethal injury on population dynamics is generally unclear, despite strong support for reduced growth and reproduction in injured individuals.

Numerous studies document negative impacts of injury on individual performance, such as reduced foraging and energy intake, and reduced energy allocated to growth and reproduction as energy is instead diverted to recovery and regeneration (reviewed by^10^). While these general patterns are fairly consistent, they are far from universal. For instance, limb loss in the freshwater crab *Paratelphusa hydrodromous* can result in faster growth, slower growth, or no change in growth rate at all depending on the timing of limb loss relative to the breeding season^11,12^. Such context-dependency appears to be the norm^10,13^ and may be driven by additional factors, such as individual age and size^14^, the severity of injury^15^, and energetic condition of the animal^16^. Despite the context-dependent nature of individual response to nonlethal injury, its pervasive influence on growth, reproduction, and future survival makes nonlethal injury an important factor in understanding population growth and dynamics.

It is particularly important to understand how nonlethal injury affects population growth and dynamics in crustaceans because this group experiences a high rate of nonlethal injury (reviewed in^17,18^), affecting nearly 30% of individuals within populations on average (reviewed in^13^). This group of consumers plays a central role in benthic^19^ and pelagic ecosystems^20^, is important economically as the target of fisheries and aquaculture^21^, and is a highly invasive taxonomic group in marine and freshwater systems around the world^22^.

Globally, considerable effort and resources are dedicated to understanding and predicting the spread, population dynamics, and impacts of invasive crustaceans and other invasive species (e.g.^23–25^). Given the pervasive impact of nonlethal injury on growth, reproduction, and survival^10,13^, nonlethal injury could play an important role in invasion dynamics if its prevalence differs across the invaded range, if its prevalence increases during important times of the year (for example, at times when energy should be allocated to reproduction), or if nonlethal injury influences growth, reproduction, or survival differently across an invasive species’ range.

The Asian shore crab *Hemigrapsus sanguineus* is an invasive species on the east coast of North America. Native to the western Pacific, it first arrived in North America in 1988 in Cape May, NJ^26^, and has experienced multiple reintroduction events since^27^. It rapidly spread from there, extending its range south to the outer banks of North Carolina and north to mid-coast Maine by 2007^28^, with its range of established populations remaining relatively stable since that time. Throughout that region, it occurs on rocky shores^29^, and in many places it has become the numerically dominant shore crab, displacing native species and the previously established invasive European green crab*, Carcinus maenas*^25,30,31^.

Within its invaded range, the Asian shore crab frequently autotomizes (i.e., self-amputates) or loses claws and walking legs^32^, possibly in response to predation attempts, entrapment, or unsuccessful molting attempts. While common, the severity of limb loss can vary widely depending on conditions, such as habitat characteristics and species interactions. For example, a previous study sampled Asian shore crabs across 20 sites from Long Island Sound to Maine and found incidences of limb loss ranging from 15.2 to 50% of individuals^33^. It should be noted that while Asian shore crabs aggressively interact with co-occurring invasive European green crabs^34,35^, aggression with conspecifics is relatively low^28,36^. Consequently, the incidence of limb loss is not influenced by Asian shore crab density alone^33^.

Regeneration of autotomized limbs is energetically expensive^13^, and in Asian shore crabs, these costs may be exacerbated by reduced amount or quality of dietary consumption. For example, a previous study found that the mass of food in the gut decreased overall with each additional limb that was missing^37^. Additionally, while Asian shore crabs readily consume mussels^38^, crabs missing a single claw during a field experiment did not consume any mussels^33^. Thus, limb loss may have negative consequences for the Asian shore crab, either by quantitatively or qualitatively altering its diet and thus energy acquisition, or by eliciting tradeoffs in energy allocation during the process of limb regeneration.

We sampled adult individuals of Asian shore crab throughout its invaded range and across an entire ‘active year’ (i.e., not including winter months) to quantify limb loss, limb regeneration, and the impact of these two factors on consumption and energy storage, growth, and reproduction. We hypothesized that limb loss and regeneration would differ across the range of this invasive species, that limb loss would alter the amount and/or quality of food consumed, and that limb loss would elicit subsequent tradeoffs between limb regeneration and the allocation of energy to storage, growth, and reproduction. If these hypothesized impacts of limb loss are supported, this would imply an important role for limb loss in the dynamics of this species in its invaded range.

## Results

Overall, we collected 799 crabs with a mean ± SD size of 21.1 ± 4.5 mm CW. Out of these 799 crabs collected in 2020 across sites, we found that 48.4% or 387 crabs were injured. Across all crabs (a total of 7990 limbs), 756 limbs were missing (9.5% of the total that could have been missing). Of these, 16.4% were claws, while the remaining 83.6% were walking legs. Of the 124 claws that were missing, 73 (58.9%) were regenerating. Out of the 632 walking legs that were missing, 403 (63.8%) were regenerating. In contrast, out of the 802 crabs collected in 2019 from New Hampshire alone, we found that just 38.7%, or 311 crabs were injured. Across all crabs from the 2019 collections (a total of 8030 limbs), 558 limbs were missing (6.9% of the total that could have been missing). Of these, 16.5% were claws, while the remaining 83.5% were walking legs. Of the 558 limbs that were missing, 362 (64.9%) were regenerating (data on the number of walking limbs versus claws that were regenerating was not collected from the 2019 samples).

### Hypothesis 1 Limb loss across sites and through time

The prevalence of limb loss (proportion of captured crabs injured) varied by site (Poisson generalized linear model that compared each of the other four sites to prevalence in a ‘reference’ site, which was Connecticut, in the center of the invaded range, *z* = − 1.97 to 2.86, *P* = 0.38 to 0.004; North to South: ME = 49.7%, NH = 36.7%, CT = 45.5%, NJ = 61.2%, NC = 62.3% of crabs injured), but did not change through time (Binomial hurdle model, *z* = − 0.28, *P* = 0.78). (The low prevalence of injury in NH may be an underestimate, as crabs without injury were preferentially sampled at this site due to miscommunication. This does not influence any metrics, other than prevalence, measured here. It should be noted that the percent of injured crabs in New Hampshire in 2020 when this sampling difference occurred is similar to the percent of injured crabs at this same site in 2019 when crabs were sampled without preference for injury status.) In addition, the number of limbs missing on injured crabs did not differ across Julian collection date (*F* = 0.0, *P* = 0.99), but did differ across sites (*F* = 4.67, *P* = 0.001), with injured crabs in Maine and New Jersey experiencing median limb loss of 2, while those in New Hampshire, Connecticut, and North Carolina had median limb loss of 1.

### Hypothesis 2 Limb regeneration across sites and through time

**Figure 1:**
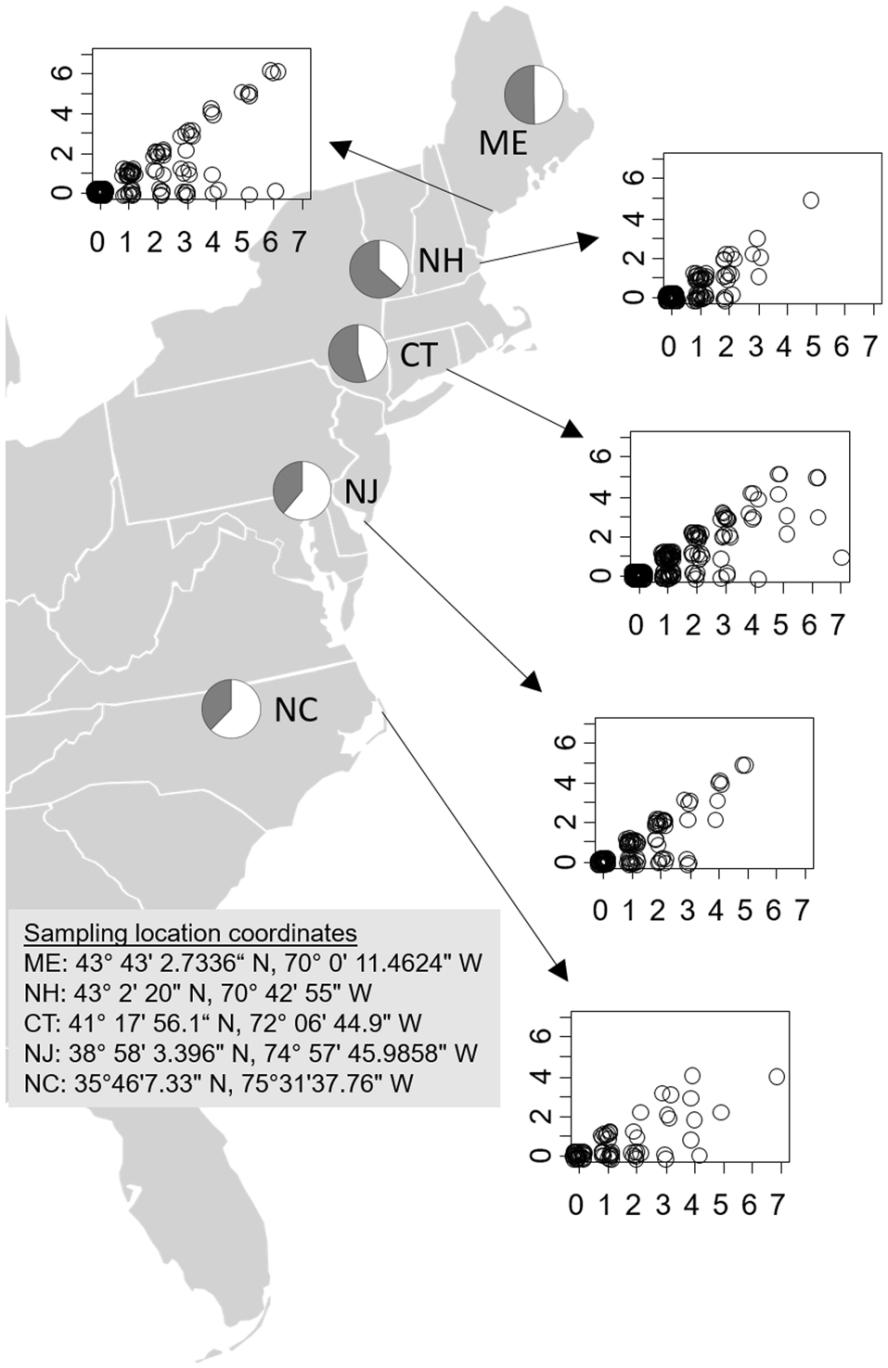
Map of the US east coast showing the five sampling sites used in 2020 along with the GPS coordinates for each sampling site. Letters on map are state abbreviations. Pie charts next to letters for each state show an increasing proportion of *Hemigrapsus sanguineus* that are injured at sites towards the southern end of the range (white = injured; gray = uninjured). The origins of the arrows pointing to each graph show the approximate locations of sampling sites. The x-axis on each inset graph is the number of missing limbs, and the y-axis on each inset graph is the number of limbs that were regenerating per crab. The data are jittered along the x-axis for clarity of presentation. Crabs missing no limbs are included in figures for completeness but were not included in statistical analyses to avoid zero-inflation.

 When the data across all sites and times were analyzed together, the number of missing limbs that were regenerating increased with the number of limbs that were missing (*z* = 14.58, *P* < 0.0001), increased weakly with Julian sampling date (*z* = 1.66, *P* = 0.097), and differed across sites (*z* = 2.49, *P* = 0.013). Specific patterns at each site are shown in Fig. 1. When each site was analyzed independently, the number of limbs regenerating at each individual site increased with the number of missing limbs (*P* < 0.0001 for all sites), with a variable frequency of non-regenerating limbs across sites. New Hampshire was the only site where limb regeneration changed through time, with the number of limbs regenerating increasing with Julian sampling date (*z* = 2.02, *P* = 0.043).

### Hypothesis 3 Impacts of limb loss on food consumed

**Figure 2:**
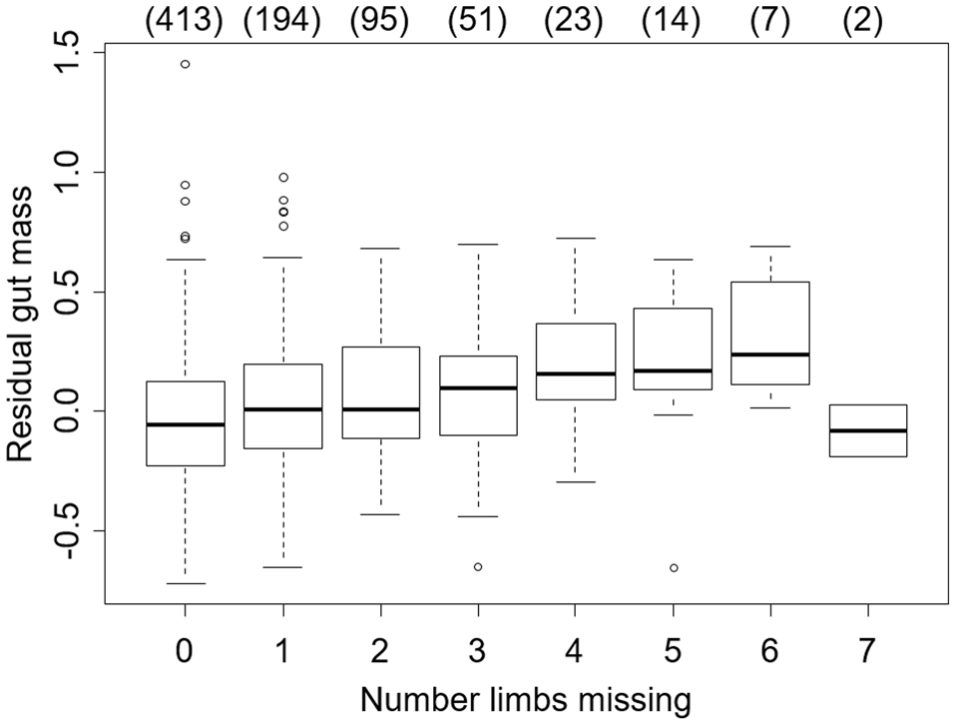
Residual gut mass, after accounting for body mass, of individual *Hemigrapsus sanguineus* sampled in 2020 as a function of the number of missing limbs. Box plots show median values (solid line), first to third quartile of the data (box), 95% of the data (whiskers), and outliers that fall outside this range (circles). Numbers in parenthesis across the top show the sample size in each category.

The residual gut mass, after accounting for differences due to body mass, increased by 48.7 ± 7.5 mg with each additional limb that was missing (*t* = 0.649, *P* < 0.0001, Fig. 2). There was no change in residual gut width with the number of limbs missing (*t* = − 0.046, *P* = 0.963), suggesting no correlation between diet quality and limb loss.

### Hypothesis 4 Impacts of limb regeneration on current and future reproductive performance

**Figure 3:**
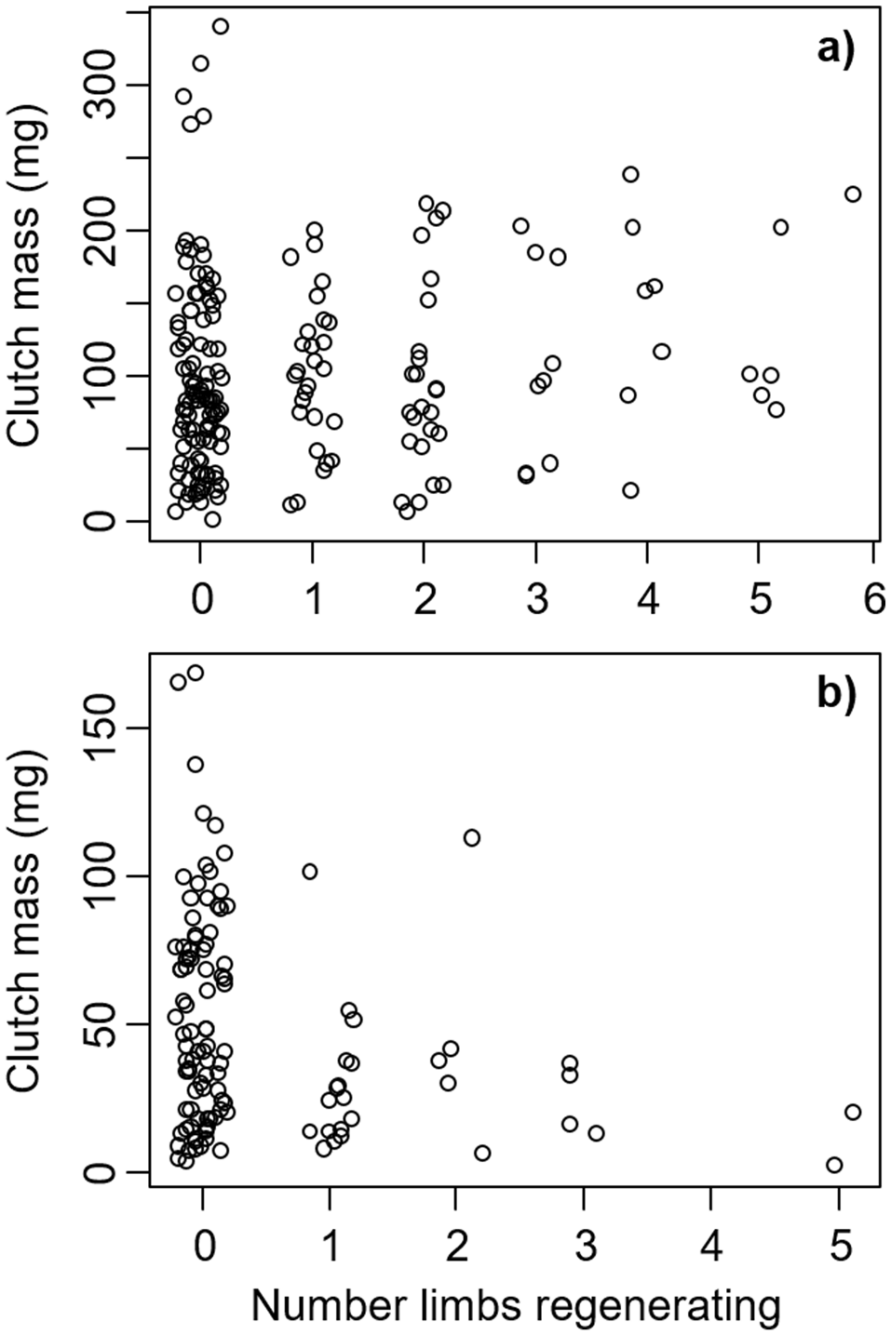
Clutch mass of *Hemigrapsus sanguineus* sampled in 2020 as a function of the number of missing limbs that are regenerating. Part (**a**) shows crabs > 20 mm CW and part (**b**) shows crabs < 20 mm CW.

**Figure 4:**
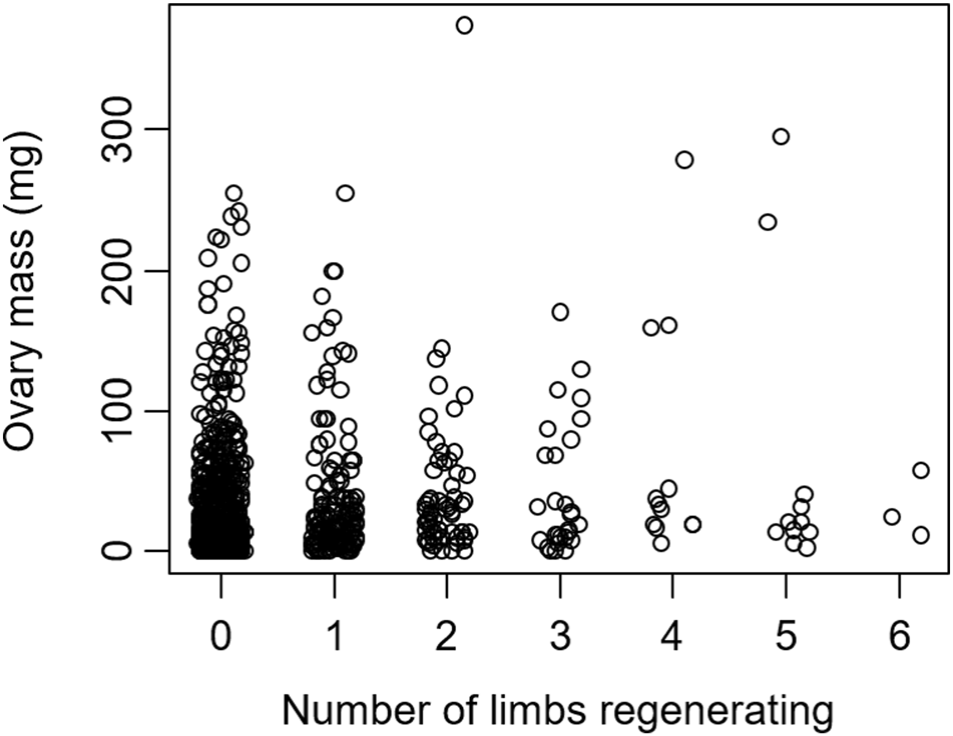
Ovary mass of individual gravid *Hemigrapsus sanguineus* of all sizes sampled in 2020 as a function of the number of missing limbs that are regenerating.

Limb regeneration had a negative impact on both current (egg mass) and future (gonad mass) reproduction. Specifically, clutch mass increased by 2.17 ± 0.46 mg for each 1-mm increase in CW (*t* = 4.68, *P* < 0.0001, Fig. 3). With each additional limb that was being regenerated, the mass of the clutch decreased by 19.75 ± 8.08 mg (*t* = − 2.45, *P* = 0.015, Fig. 3). Additionally, the interaction between CW and limb regeneration was significant (*t* = 2.98, *P* = 0.003), indicating that the impacts of limb regeneration on clutch size depended on crab size. Specifically, clutch size increased weakly with the number of limbs that were regenerating for crabs that were larger (> ~ 20 mm CW, Fig. 3a), while clutch size declined strongly with the number of regenerating limbs for crabs that were smaller (< ~ 20 mm CW, Fig. 3b), though it should be noted that this is based on a small sample of crabs < ~ 20 mm CW that were regenerating multiple limbs. These reductions in clutch mass with limb regeneration resulted from reductions in individual egg size, rather than changes in egg number. Specifically, egg diameter declined by 0.0023 ± 0.0012 mm with each lost limb that was regenerating (*t* = − 1.955, *P* = 0.052), while the number of eggs in the clutch did not change with limb regeneration (*t* = − 0.343, *P* = 0.732). Similarly, using 2019 data for female crabs, ovary mass increased by 15.36 ± 1.41 mg with each 1-mm increase in CW (*t* = 10.89, *P* < 0.0001), and with each additional regenerating limb, the ovary mass decreased by 13.77 ± 3.78 mg (*t* = − 3.64, *P* = 0.0003). For male 2019 crabs, testes mass increased by 1.15 ± 0.21 mg with each 1-mm increase in CW (*t* = 5.54, *P* < 0.0001), and with each additional regenerating limb, the testes mass decreased by 3.09 ± 1.27 mg (*t* = − 2.44, *P* = 0.017). Results from 2020 females were similar, as ovary mass increased by 4.94 ± 0.36 mg with each 1-mm increase in CW (*t* = 13.91, *P* < 0.0001), and decreased by 19.72 ± 6.20 mg with each additional limb that was regenerating (*t* = − 3.18, *P* = 0.002, Fig. 4). Additionally, the interaction between CW and limb regeneration was significant (*t* = 3.19, *P* = 0.001), indicating that the impacts of limb regeneration on ovary mass depended on crab size.

### Hypothesis 5 Impacts of limb regeneration on energy storage

The mass of the hepatopancreas increased by 10.45 ± 3.10 mg for each 1-mm increase in CW (*t* = 33.72, *P* < 0.0001). In contrast, energy storage in the hepatopancreas was not influenced by the number of regenerating limbs (*t* = − 1.11, *P* = 0.265).

### Hypothesis 6 Impacts of limb regeneration on growth

**Figure 5:**
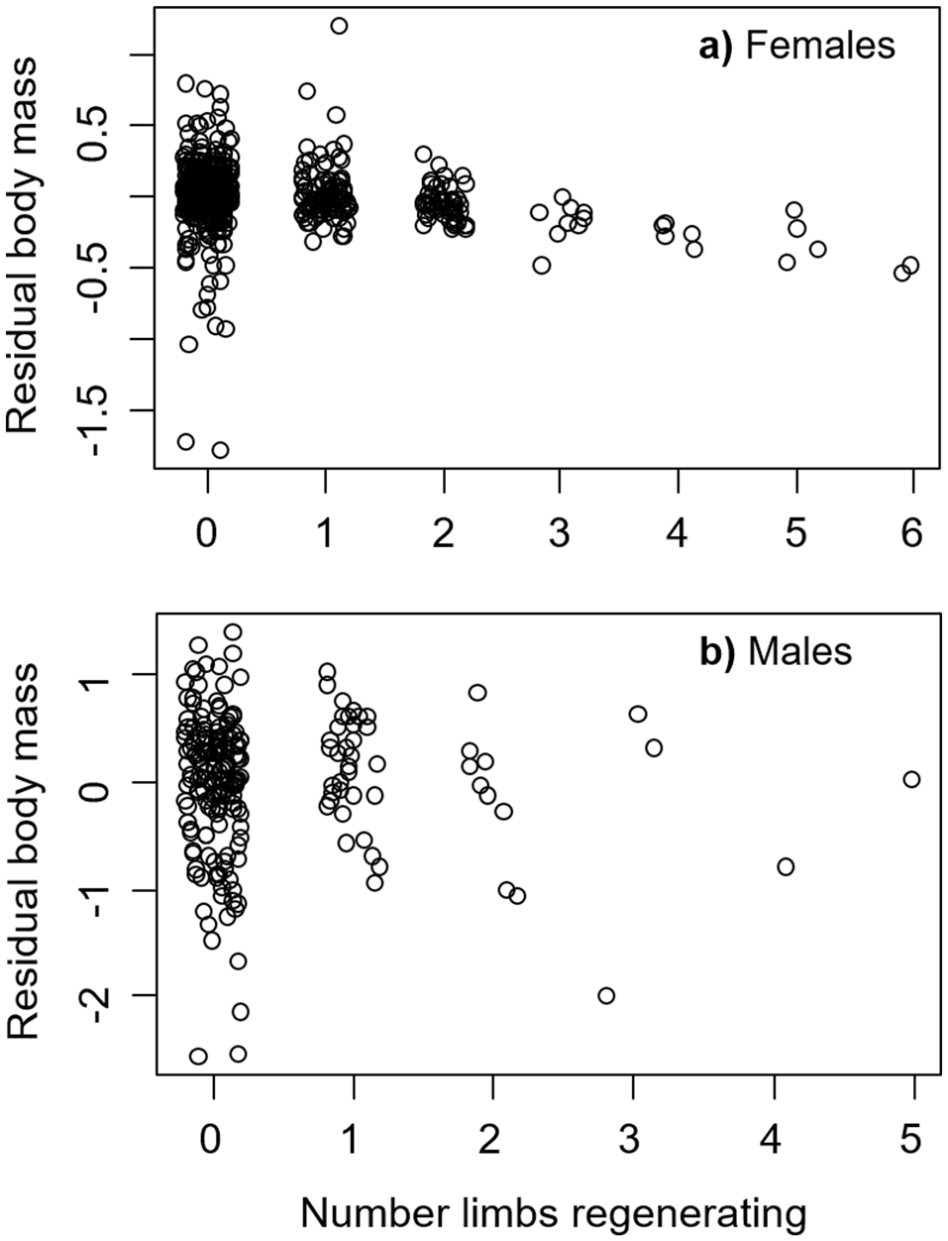
Changes in residual body mass (after accounting for differences with CW) of individual female (**a**) and male (**b**) *Hemigrapsus sanguineus* sampled in 2019 with the number of missing limbs that are regenerating.

Based on data from the 2019 sampling at New Hampshire, there was no difference in the number of limbs missing between male and female crabs (*t* = 0.25, *P* = 0.805). However, there was a difference in the number of limbs regenerating, with females regenerating 72.5% of missing limbs, and males regenerating just 45.2% of missing limbs (*t* = 2.81, *P* = 0.005). For female crabs, we found that, after accounting for changes in body mass (minus the ovary and hepatopancreas) with CW, body mass decreased by 48.7 ± 9.8 mg with each limb that was regenerating (*t* = − 4.94, *P* < 0.0001, Fig. 5a). For males, the impact on growth was even stronger. We found that residual body mass decreased by 143.0 ± 59.8 mg with each regenerating limb (*t* = − 2.39, *P* = 0.019, Fig. 5b), though this result is based on a relatively small number of male crabs that were regenerating multiple limbs.

### Hypothesis 7 Impacts of size and sex on limb regeneration after limb loss

**Figure 6:**
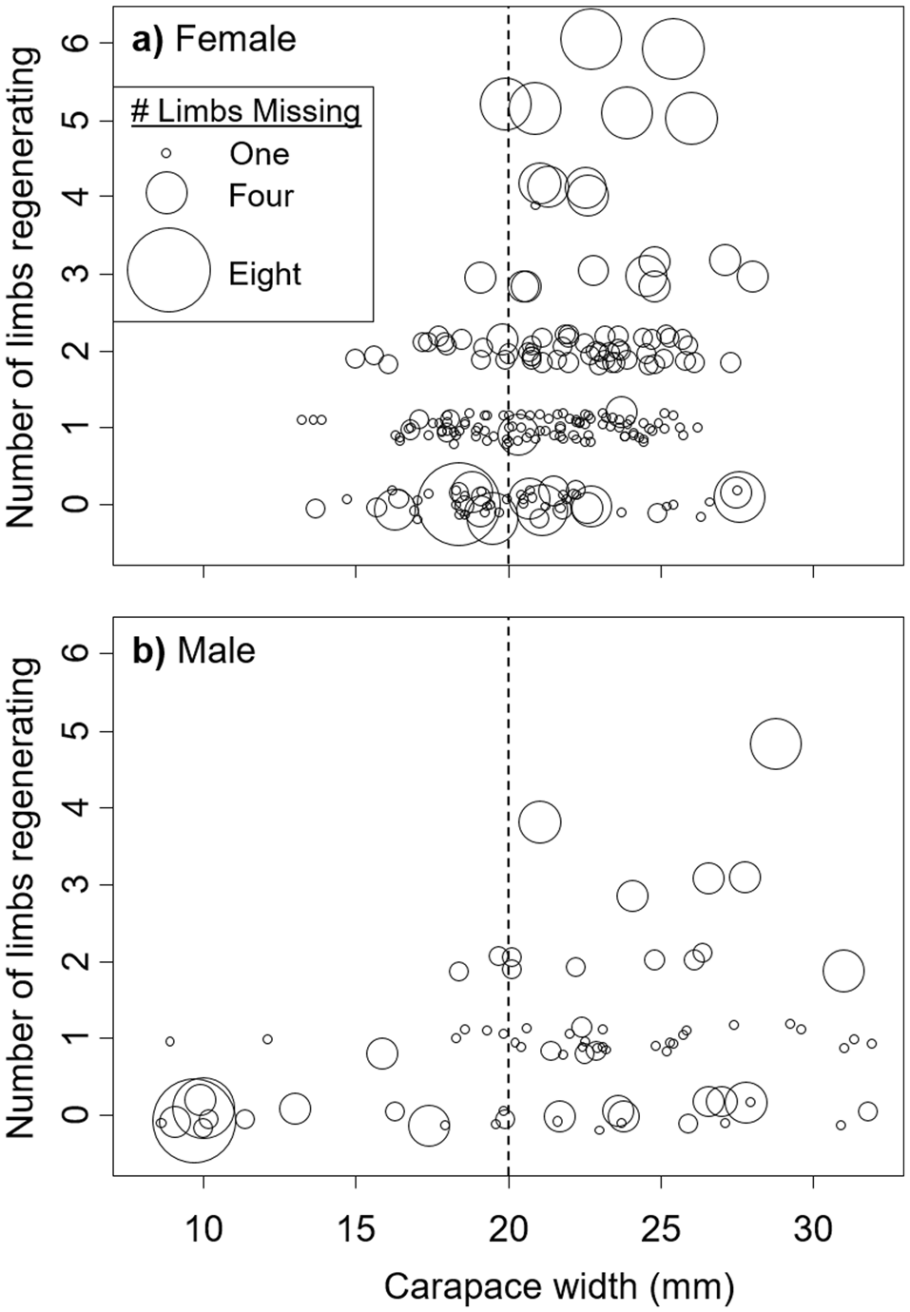
Number of limbs regenerating for female (**a**) and male (**b**) *Hemigrapsus sanguineus* from the 2019 sampling in New Hampshire as a function of CW. Circle size shows the relative number of missing limbs, as indicated in the inset box. The dashed vertical line shows the cutoff used in the analysis to examine crabs < 20 mm or ≥ 20 mm CW.

Our results suggest a size-specific change in energy allocation following injury in *Hemigrapsus sanguineus*. Specifically, based on crabs from the 2019 sampling, we found that the number of limbs regenerating in crabs < 20 mm CW was not influenced by the number of missing limbs (female analysis: *z* = 1.50, *P* = 0.13, male analysis: *z* = − 1.35, *P* = 0.18, circle size does not increase with height to the left of the dashed line in Fig. 6), while the number of limbs regenerating in crabs ≥ 20 mm CW increased with the number of missing limbs (female analysis: *z* = 7.59, *P* < 0.0001, male analysis: *z* = 3.30, *P* = 0.001, circle size increases with height to the right of the dashed line in Fig. 6).

## Discussion

Nonlethal injury is a common phenomenon in animal systems, and often has important implications for vital rates that underlie population dynamics, including growth, reproduction, and survival. We examined limb loss in the invasive Asian shore crab *H. sanguineus* to determine the extent to which this nonlethal injury influences vital rates throughout its invasive North American range. We have shown that the prevalence of limb loss in Asian shore crabs is generally higher towards the southern end of its range than in the northern end (H1) and that limb regeneration differs across sites, sometimes in nonlinear ways throughout the active (non-winter) months of the year (H2). We have also shown that injured crabs appear to consume more, presumably in an effort to finance the extra energetic costs of limb regeneration (H3). Despite this, injured crabs that are regenerating limbs suffer reduced current and future reproduction, resulting in the production of smaller eggs (H4). In addition, energy storage was not influenced by limb regeneration (H5), but body mass decreased with increasing effort towards limb regeneration, suggesting lower growth rates (H6). Finally, our data suggest that smaller crabs allocate less energy towards limb regeneration than larger crabs (H7).

Prevalences of injury reported here are similar to levels previously documented for this species. Davis et al.^32^ sampled crabs from Connecticut and found that 42% were missing at least one limb, compared to our finding of 46% of crabs in Connecticut. Similarly, Vernier and Griffen^37^ found that 51% of crabs in New Hampshire were injured, compared to 37% reported here for the same site. Delaney et al.^33^ reported that across 20 sites in New England, 31% of *H. sanguineus* were missing at least one limb, compared to 43.9% of crabs in our three New England sites (ME, NH, CT) combined.

Despite the documented success of Asian shore crabs as an invader, results here suggest that injury may be one factor working against its success. A review of injury in decapod crustaceans concluded that the impacts of limb loss on survival in the field are relatively unknown, but that it likely depends on whether limb loss occurs cleanly via autotomy on the breakage plane or whether limb loss leaves an open wound that leads to loss of hemolymph^13,39^. This same review found that published reports of injury reveal ~ 30% prevalence on average in decapod populations. This is considerably lower than prevalences reported here for sites spanning much of the Asian shore crabs’ range. High prevalence of injury in living individuals suggests that limb loss may not substantially increase mortality risk for individual Asian shore crabs.

While injury may not increase mortality in this invader, our results demonstrate strong impacts of injury on individual energetics. In contrast to Vernier and Griffen^37^, we found that food consumption increased with limb loss. The reason for this discrepancy is not clear, but could reflect differences across sites or times of year sampled here, as opposed to samples collected at a single time and only in New Hampshire by Vernier and Griffen^37^. Increased consumption following injury, based here on gut mass, is in contrast to mechanistic changes in consumption following injury that have previously been reported. Davis et al.^32^ showed that injured crabs fed more slowly than noninjured crabs, and Delaney et al.^33^ showed that injured crabs did not eat mussels that were readily consumed by noninjured crabs. The discrepancy is likely because both of these previous studies examined the impact of claw loss specifically. We found that the majority of injury observed was loss of walking legs, which is likely to impose minimal, if any, limitations on food consumption. Increased food consumption is presumably an attempt to meet the extra energetic costs of limb regeneration; however, observed reduced growth and reproduction suggest that this increased food consumption was insufficient to entirely meet the extra energetic needs.

We found that limb regeneration resulted in ~ 77 mg lower residual body mass, after accounting for CW, for each regenerating limb. Depending on the size of the crab, this is approximately equivalent to the weight difference of crabs that differ in CW by ~ 0.5 mm. Thus, as a rough approximation, we may expect each limb to reduce the growth increment at the next molt by approximately 0.5 mm. Alternatively, if crabs molt once a set amount of tissue growth has been achieved, injury would lengthen the molt interval rather than decrease the growth increment. Either one of these possibilities would reduce the overall growth rate of the individual. Due to the greater mass of larger crabs, we should expect this decrease in growth to be more pronounced in smaller crabs. For instance, a crab of 26 mm CW has a mass that is ~ 235 mg greater on average than a crab that is 25 mm CW, while a crab of 16 mm CW has a mass that is just 115 mg greater than a crab that is 15 mm CW. Thus, the mass reduction from losing a single limb (~ 77 mg) is equivalent to 0.66 mm growth for a 15 mm crab, but is equivalent to just 0.33 mm growth for a 25 mm crab. This difference may explain the difference we found in limb regeneration strategy for small and large crabs, where large crabs (≥ 20 mm CW) were much more likely to allocate energy to limb regeneration than were small crabs.

Reproduction in crabs is strongly size-dependent, increasing via positive allometry with CW^40^. For Asian shore crabs captured here, egg mass increased allometrically with CW according to the equation *aCW*^*b*^, where *b* = 1.84. Thus, a small crab stands to lose much more reproductive potential by allocating energy to limb regeneration (i.e., it loses more growth at the next molt) than does a larger crab. Our findings also show that limb regeneration has large detrimental impacts on current reproduction (clutch size) for small crabs, but not for large crabs. As a result, smaller crabs should be expected to forgo limb regeneration in favor of current reproduction and growth in order to maximize future, and thus lifetime, fecundity. Thus, there appears to be a size-dependent growth-regeneration trade-off, driven by both current and future reproductive potential.

In addition to decreasing growth, and thus size-dependent fecundity, limb regeneration further negatively influenced reproduction through direct reduction of egg production. Specifically, we found that the egg mass of gravid crabs decreased by ~ 20 mg for each additional limb being regenerated, for crabs of the same size. This impact presumably stems from a relocation of energy away from egg production and towards regeneration, increasing the trade-off costs to reproduction.

We have examined a snapshot of the impacts of injury on individual animals across a short time, but these momentary impacts will also extend through time to have longer implications. For instance, injury that reduces growth rate will reduce lifetime fecundity due to the size-dependent reproductive potential of crabs. Similarly, impacts on a single clutch demonstrated here may extend to impacts on multiple clutches, depending on the timing of injury relative to molting and reproduction. These extended individual impacts may further scale up to population level consequences, such as reduced population growth rates. This scaling up process depends on population density and size structure and may be mediated by other factors such as food availability. Such numerical extrapolations are beyond the scope of this study. Nevertheless, the increased prevalence of injury towards the southern end of its invaded range suggests that this is where injury may have its greatest impact on populations, and thus on the invasion success of Asian shore crabs. Increased injury towards the southern end of the range may reflect warmer temperatures in the south that lead to increased metabolic costs for this poikilotherm, and thus likely reduce its energetic scope for growth needed for regeneration^41,42^. This temperature-induced reduction in scope for growth may further interact with the negative consequences of nonlethal injury documented here to amplify the negative population impacts (i.e., reduced growth and reproduction) of nonlethal injury. Alternatively, increased injury towards the southern portion of the invaded range may reflect limited refuge habitat that provides protection from predation. Additional research is needed to identify the relative importance of these alternative possible reasons for latitudinal differences in the prevalence of injury.

The prevalence of injury is high across many types of organisms^4^, and the frequency of injury may be increasing as human impacts on natural systems intensify^6–8^. Here, we have provided an example of the broad suite of impacts from nonlethal injury for one species that is a widespread coastal invader. Similar impacts and tradeoffs from nonlethal injury have recently been shown for other species^43^. This suite of injury impacts, and others not measured here, likely play out amongst invasive and native species across most or all habitat types. Explicitly accounting for nonlethal injury in ecological studies may therefore be important in accounting for a major source of energetic variation within populations that influences nearly all aspects of individual, and thus of population, performance.

## Methods

### Sampling

We sampled crabs from 5 collection sites, including Bailey Island in Harpswell, Maine; Rye, New Hampshire; Goshen Point at Harkness Memorial State Park in Waterford, Connecticut; Cape May Ferry Park, North Cape May, New Jersey; and Oregon Inlet, North Carolina (Fig. 1). Sites were chosen to represent a relatively even spatial distribution over more than 1000 km of coastline across the species’ invaded range, at sites where sufficient Asian shore crab populations existed to facilitate repeated sampling throughout the study (based on our prior knowledge of these sites).

We sampled during days surrounding spring tide periods around the middle of March, May, July, September, and November 2020. We chose this sampling interval to encompass the reproductive season of this species^29,44^. In contrast to this general sampling, we chose to sample monthly throughout this same period at Connecticut, the site in the middle of the invaded range, in order to provide a more complete picture of temporal variation within a single site.

Asian shore crabs forage most intensively during nighttime high tides^45,46^. At each location and sampling period, crabs were therefore collected by hand during morning low tides from low- to mid-intertidal rocky shores to maximize the likelihood of food in the guts. We collected ~ 30 adult females (individuals > 12 mm CW^44^) at each sampling from each site (n = 799 total), with at least 20 of these being gravid during May, July, and September sampling dates. We focused on females to facilitate exploring the effects of limb loss on reproduction. At all sites except New Hampshire, samples were collected based on occurrence and without respect to injury status; however, collections in New Hampshire preferentially excluded injured individuals (see “Results” for implications of this difference). Upon collection, crabs were placed in individual small sample bags to ensure that any limbs lost post-capture were kept with the crab for body mass assessments and were not counted as injured in analyses. Each crab was frozen upon collection. All samples were then shipped on dry ice to Brigham Young University in Provo, UT where they were stored at − 80 °C until dissection.

In addition to the samples described above, we also include a second set of data taken from crabs that were collected from the same site in New Hampshire (Odiorne Point State Park) during July 2019. This second dataset was included because it is a large sampling of both females (n = 585) and males (n = 217) that were all collected at the same place and time and therefore avoids any complicating impacts of spatial and temporal differences. Samples were transported and stored in the same way as described above.

### Dissections

All crabs described above were processed in the same manner, with the exception that the egg masses were measured only for crabs sampled in 2020. Crabs were dissected by first bringing them to room temperature by immersing the individual sampling bags in room temperature water. We then measured carapace width (CW) to the nearest 0.5 mm using a Vernier caliper and counted the number of missing limbs and the number of limbs that were regenerating, based on the presence of limb buds. For gravid crabs (from 2020 only), we then separated the clutch from the pleopods following methods given by Choy^47^. Specifically, we removed the individual pleopods with attached eggs and submerged and agitated them in a 6% sodium hypochlorite bleach for 1 min 15 s. We then added a generous amount of 3% sodium thiosulfate to neutralize the bleach. Separated eggs were then immediately rinsed with deionized water to remove hypochlorite and thiosulfate solutions and were then immediately returned to saltwater and allowed ≥ 30 min to revert from any potential volume changes that may have occurred from osmosis during the bleach, sodium thiosulfate, and deionized water treatments. Egg samples were then photographed under microscopy using an Olympus MVX10 microscope (model SZX-ILLB100) with high-resolution digital camera (Olympus DP74) and cellSens imaging software. After this, we again rinsed eggs with deionized water to remove any salt and placed the eggs into a pre-weighed aluminum drying boat, and dried them to constant weight at 60 °C by checking them daily until changes in mass from day-to-day were < 0.00002 g. Salt was removed because, if included, its mass may have overestimated the small mass of the egg samples. The microscope photographs were processed using ImageJ software (following^48^) to count the number of eggs in each clutch and to measure the diameter of up to 10 eggs from each clutch using only eggs that were in the initial stage of development.

Next, we dissected each crab by removing the dorsal carapace. We then extracted the cardiac stomach, the ovary, and the hepatopancreas and placed each into separate pre-weighed drying boats. All other parts of the crab were placed into another pre-weighed drying boat and each part of the crab body was then dried to constant weight at 60 °C, as above. Each was then weighed using a Mettler Toledo DualRange scale (model #XS205). To estimate diet quality, we measured the width of the pre-dried cardiac stomach, since higher quality diets in crabs, and in this species in particular, result in smaller gut widths^49^.

### Statistical tests of hypotheses

#### Hypothesis 1 Limb loss across sites and through time

To test whether the prevalence of limb loss remains constant across sites and through time, we implemented a “hurdle” or “two-stage” analysis^50^, using only the 2020 dataset collected across latitude. We used a hurdle analysis to avoid zero inflation due to the large number of uninjured crabs. To do this, we first conducted a generalized linear model with a binomial distribution on whether crabs were injured or not as a function of the collection site and Julian sampling date. This was followed by a general linear model using only data for crabs that had an injury (i.e., eliminating all zero injuries), with the number of limbs missing as the response and collection site and Julian sampling date as predictors, followed by a Tukey’s HSD test for differences between sites.

#### Hypothesis 2 Limb regeneration across sites and through time

To test whether limb regeneration following limb loss differs across sites or through time, we used a generalized linear model with a Poisson distribution for count data, with the number of limbs regenerating as the response variable, and the number of limbs missing, Julian sampling date, and collection site as predictor variables. Given the large number of uninjured crabs, only crabs with missing limbs were included in this analysis to avoid zero inflation. This was followed by individual generalized linear models, again with Poisson distributions, for each site, with the number of limbs regenerating as the response and the number of limbs missing and Julian sampling date as the predictor variables. These analyses again only included injured crabs.

#### Hypothesis 3 Impacts of limb loss on food consumed

To test the effect of limb loss on the amount or quality of food consumed, we pooled all the sampling data across sites and through time (only using 2020 samples; similar analyses and results were obtained from the 2019 samples in New Hampshire, but are not reported). We first regressed log gut mass on log body mass and determined the residuals from this analysis. We then used this residual gut mass after accounting for body mass as the response variable in a linear model with the number of limbs missing as the predictor variable. We accounted for body mass using residuals because larger crabs are expected to consume more food. We determined whether diet quality differed with injury by examining residual gut width (after accounting for CW) using a linear model, with the number of limbs missing, Julian sampling date, and collection site as predictors.

#### Hypothesis 4 Impacts of limb regeneration on current and future reproductive performance

To test the effect of limb regeneration on current or future reproductive performance across the species range, we analyzed 2019 and 2020 data separately. We examined the impacts of limb regeneration on current reproductive effort using 2020 data only (since egg data were not available for 2019). We used a linear model to analyze changes in egg mass as a function of CW and the number of limbs that were regenerating. To determine whether egg mass changes resulted from fewer eggs or smaller eggs, we also ran two additional linear models, one with total clutch size as the response variable and one with egg size as the response variable. We examined the impact of limb regeneration on future reproduction using both 2019 and 2020 data, analyzed separately. For crabs sampled in 2019, we used linear models for males and females separately to analyze changes in gonad mass as a function of CW and the number of limbs regenerating, using only crabs that were missing at least one limb. For crabs sampled in 2020, we used an identical approach. We initially analyzed individual sites separately, but the results were all qualitatively similar, so we pooled the data across sites and sampling dates for this analysis.

#### Hypothesis 5 Impacts of limb regeneration on energy storage

To test whether limb loss or regeneration influence energy storage across sites, we used a linear model with hepatopancreas mass as the response variable and with CW and the number of limbs regenerating treated as predictor variables with data pooled across sites and sampling dates from 2020. In addition, individual sites were analyzed separately, but results were all qualitatively similar, and so data were pooled for analysis.

#### Hypothesis 6 Impacts of limb regeneration on growth

To test whether limb loss influences growth, we examined males and females separately using data from crabs collected in 2019. On a primarily herbivorous diet that is expected for Asian shore crabs, differences in body mass reflecting growth can be quite small^51^, and therefore require minimal noise to detect a signal. We therefore used the 2019 data to remove any possible impacts of latitudinal or temporal differences. We first compared the number of limbs missing and the number of limbs regenerating for the two sexes using separate *t* tests. Body mass may change for a given size (CW) of crab due to changes in reproductive effort (gonad mass), changes in energy storage (hepatopancreas mass), or changes in muscle mass (i.e., growth). To isolate growth, we therefore subtracted gonad mass and hepatopancreas mass from total body mass. Next, for each sex, we first determined the allometric relationship between this modified body mass and CW by fitting the nonlinear equation *mass* = *a* × *CW*^*b*^ to the data. We then determined the residuals from this relationship and used the residual body mass after accounting for differences in CW as the response variable in a linear model with number of limbs regenerating as the predictor variable, and only using data from crabs where at least one limb was missing.

#### Hypothesis 7 Impacts of size and sex on limb regeneration after limb loss

To test whether limb regeneration following limb loss differs with the size or sex of the crab, we used the 2019 dataset collected from only New Hampshire. We analyzed these data for females and males separately. Graphical inspection of the data suggested that 20 mm CW might represent a transition point where crabs above and below this size responded differently to limb loss. We therefore conducted the same analysis for each sex on crabs below 20 mm CW and above 20 mm CW. For each, we conducted a generalized linear model with a Poisson distribution for count data on the number of limbs regenerating, with the number of limbs missing treated as the independent variable. These analyses only included crabs where at least one limb was missing.

## Data Availability

Data presented here will be deposited on Dryad once the paper is accepted for publication.
